# Unimolecular Solvolyses in Ionic Liquid: Alcohol Dual Solvent Systems

**DOI:** 10.3390/molecules21010060

**Published:** 2016-01-06

**Authors:** Elizabeth D. Kochly, Nicole Jean Lemon, Anne Marie Deh-Lee

**Affiliations:** Department of Chemistry, Mills College, 5000 MacArthur Blvd, Oakland, CA 94613, USA; nlemon@mills.edu (N.J.L.); adehlee@mills.edu (A.M.D.-L.)

**Keywords:** ionic liquids, organic synthesis, green chemistry, solvolysis, carbocation, nucleophile, dual solvent, Kamlet–Taft parameters, hydrogen bonding

## Abstract

A study was undertaken of the solvolysis of pivaloyl triflate in a variety of ionic liquid:alcohol solvent mixtures. The solvolysis is a k_Δ_ process (*i.e.*, a process in which ionization occurs with rearrangement), and the resulting rearranged carbocation intermediate reacts with the alcohol cosolvent via two competing pathways: nucleophilic attack or elimination of a proton. Five different ionic liquids and three different alcohol cosolvents were investigated to give a total of fifteen dual solvent systems. ^1^H-NMR analysis was used to determine relative amounts of elimination and substitution products. It was found, not surprisingly, that increasing the bulkiness of alcohol cosolvent led to increased elimination product. The change in the amount of elimination product with increasing ionic liquid concentration, however, varied greatly between ionic liquids. These differences correlate strongly, though not completely, to the Kamlet–Taft solvatochromic parameters of the hydrogen bond donating and accepting ability of the solvent systems. An additional factor playing into these differences is the bulkiness of the ionic liquid anion.

## 1. Introduction

Interest in ionic liquids (ILs) as solvents has steadily grown over the past two decades. Researchers have shown that ILs support a wide variety of reactions types and, in the process, have discovered many advantages and disadvantages to using these solvents. Quite frequently, however, differences are seen between reactions occurring in IL solvents and the same reactions run in “traditional” solvents. Sometimes, these differences can be quite advantageous and/or quite interesting. However, of course, they lead to even more questions about the fundamental nature of these designer solvents. A thorough understanding of how ILs behave and why is key to using them efficiently.

Nucleophilic substitution reactions are one such instance of different and unexpected behavior in ILs. Bimolecular substitution reactions in particular have received a lot of attention in the literature. The lab of Kim developed an efficient bimolecular substitution (S_N_2) fluorination method using potassium fluoride (KF) in ILs [1] and then expanded upon this work to incorporate a variety of other nucleophiles, including water [2,3]. Welton extensively studied the effects of ionic liquids on solute nucleophilicity in S_N_2 reactions, correlating them to Hughes–Ingold rules of the solvent effects and Kamlet–Taft solvatochromic parameters for hydrogen bonding [4,5,6,7]. Comprehensive reviews have been published by Lancaster [8] and Hallett and Welton [9]. More recently, the Harper group has undertaken more detailed studies of the effects of individually varying the IL cation [10] and the IL anion [11] on S_N_2 reactivity. Their work has shown that the identities of both the IL cation and anion have little effect on the reaction outcome other than general electrostatic effects.

Less well studied are the unimolecular substitution reactions: those involving carbocation intermediates. Our earliest study demonstrated that a variety of carbocations readily form in ILs and subsequently react with trace amounts of water present (due the hygroscopic nature of ILs) [12]. Chiappe used carbocation decay measurements to show that carbocations have shorter lifetimes and therefore appear to be more electrophilic in ILs [13]. Harper then published a unimolecular solvolysis study using alcohol cosolvents and demonstrated that both the rate of solvolysis and the enantiomeric excess of the products varied with IL concentration [14].

Our group has recently been interested in how an ionic liquid solvent affects the competing pathways of nucleophilic attack and proton elimination in unimolecular solvolysis reactions. Our preliminary studies have shown that increasing the mole fraction of IL in an IL:alcohol dual solvent system increases the favorability of the elimination pathway *vs.* nucleophilic attack [15,16]. It was observed that nearly all IL cosolvents caused an increase in the favorability of the elimination pathway. In addition, variations of this effect among different ILs could be correlated to Kamlet–Taft solvatochromic parameters of the hydrogen bond donating (α) and accepting (β) ability of the ionic liquid cosolvent. These factors have an impact on the reactivity of both the alcohol (*i.e.*, nucleophilicity *vs.* basicity) and the carbocation intermediate. Since the cationic component of the IL is primarily responsible for the α value and the anionic component of the IL is primarily responsible for the β value, the degree of favorability of the elimination pathway can be fine-tuned by careful selection of the IL components. The current study expands upon this research by widening the work to include a variety of additional alcohol cosolvents in an effort to understand the impact of steric and electronic effects of the alcohol on this competition.

## 2. Results and Discussion

**Scheme 1:**
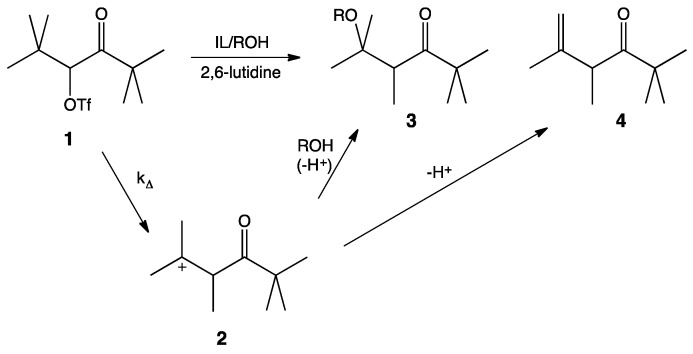
Mechanism of the solvolysis of α-keto triflate **1**.

It is widely accepted that solvolysis of α-keto triflate, **1**, follows a k_∆_ mechanism in which loss of the leaving group occurs in concert with a methyl shift to give the more stable tertiary carbocation, **2**. Nucleophilic attack by the protic cosolvent (*i.e.*, HOS) leads to the substitution product, **3**, and deprotonation by the cosolvent leads to the elimination product, **4** (Scheme 1). 2,6-Lutidine is a non-nucleophilic base that acts as an acid sponge to absorb the equivalent of trifluoromethanesulfonic acid produced during the reaction.

As mentioned, previous unimolecular studies have used kinetics as an investigative tool. While highly useful and informative, kinetics can only give information about the rate-limiting step of a reaction, *i.e.*, the formation of the carbocation. Our interest lies in the second step of this mechanism: What affect does the IL have on the interaction between nucleophile and carbocation? An analysis of the product ratios gives information about this competition between nucleophilic attack and proton elimination. Product ratios for this reaction are easily determined by ^1^H-NMR integration of the α-carbonyl proton of each product in the crude product mixture.

**Figure 1:**
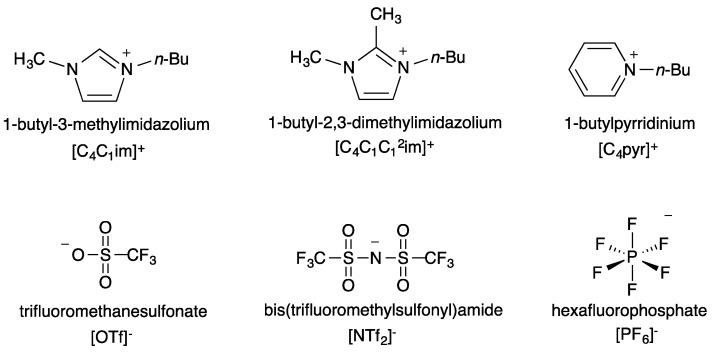
Structures and abbreviations of the cationic and anionic IL components chosen for this study.

One of the main advantages of ILs is that their properties can be fine-tuned by the careful selection of cationic and anionic components. Making use of this, we studied three ILs for which the cationic component is identical and the anion varied and three ILs for which the anionic component is identical and the cation varied. Structures and abbreviations for the cations and anions are seen in Figure 1.

**Figure 2:**
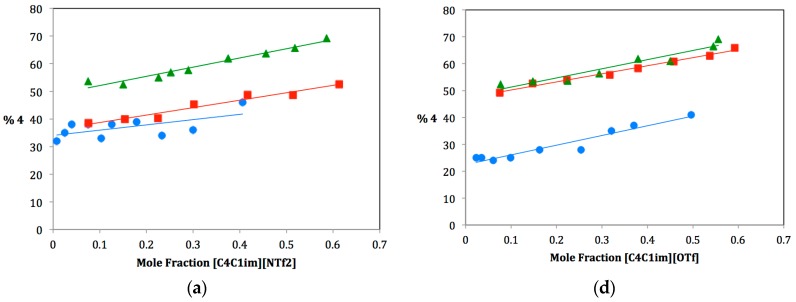

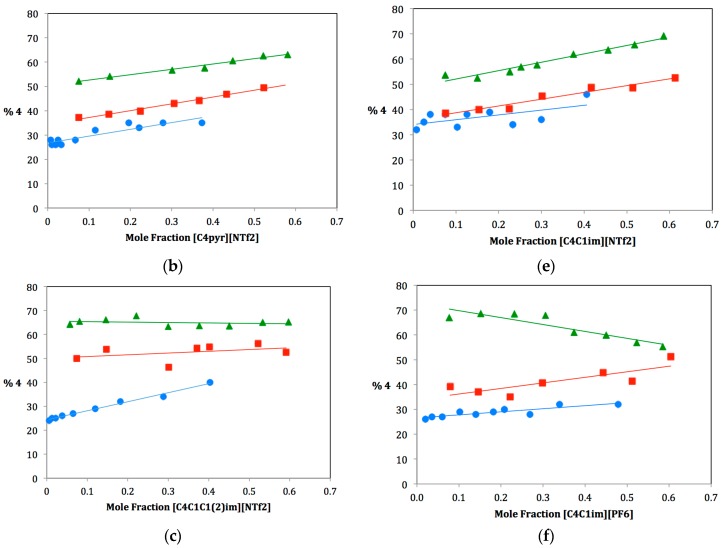
Plots of the percentage of elimination Product **4** *vs.* mole fraction of IL for methanol (•), ethanol (▪) and isopropanol (▲).

As ILs tend to be hygroscopic to varying degrees, great care was taken to ensure that each IL used be extremely dry. Three anhydrous protic cosolvents were chosen for the study: methanol, ethanol and isopropanol. For each dual-solvent system, the mole fraction of IL was varied from near zero to approximately 0.6 and plotted against the percentage of elimination product produced, **4**. It was not feasible to study mole fractions of greater than 0.6 due to the large difference in the molecular weight of the ILs and the alcohols. A large mole fraction of IL would require such a small amount of alcohol cosolvent that measuring the alcohol to the exclusion of water was impractical. Plots for the fifteen solvent systems are shown in Figure 2. The ILs have been divided into two series: three ILs on the left (a, b, c) have the same anionic component and different cationic components, three ILs on the right (d, e, f) have the same cationic component and different anionic components. Note that the plot for [C_4_C_1_im][NTf_2_] is shown twice since it belongs to both series. For each dual–solvent system, the slope is a correlation of the rate of change of Product **4** with increasing mole fraction of IL. Comparing these slopes can therefore tell us much about how different solvent systems affect the preferred solvolysis pathway. Slopes are summarized in Table 1 along with the Kamlet–Taft solvatochromic parameters for hydrogen bond donating and accepting ability, α and β, respectively [17].

**Table 1:** Rate of change of **4** with increasing mole fraction of IL for each dual-solvent system (*i.e.*, slope (error)) and Kamlet–Taft (KT) solvatochromic parameters for each IL and protic solvent [17].

**Entry**	**Ionic Liquid and KT Parameters**	**CH_3_OH_16_**	**CH_3_CH_2_OH**	**(CH_3_)_2_CHOH**
		α = 1.00	α = 0.86	α = 0.76
		β = 0.70	β = 0.80	β = 0.84
(a)	[C_4_C_1_im][NTf_2_] α = 0.62, β = 0.24	19.1 (8.48)	26.9 (2.93)	33.3 (2.87)
(b)	[C_4_pyr][NTf_2_] α = 0.54, β = 0.25	27.8 (4.09)	28.0 (1.41)	22.0 (1.44)
(c)	[C_4_C_1_C_1_^2^im][NTf_2_] α = 0.38, β = 0.24	37.7 (1.60)	7.34 (7.53)	−1.78 (2.76)
(d)	[C_4_C_1_im][OTf] α = 0.63, β = 0.46	36.1 (3.76)	30.1 (1.69)	34.1 (3.91)
(e)	[C_4_C_1_im][NTf_2_] α = 0.62, β = 0.24	19.1 (8.48)	26.9 (2.93)	33.3 (2.87)
(f)	[C_4_C_1_im][PF_6_] α = 0.63, β = 0.21	12.4 (2.33)	22.4 (7.18)	−27.9 (5.45)

The data for IL methanol mixtures came from our previous study in which we investigated how differences in the α and β values of the ILs affected the product ratio [16]. This current study expands the dataset by including additional alcohol cosolvents to determine whether the effects previously described are consistent among other IL alcohol dual solvent systems. The selection of alcohol solvents (methanol, ethanol, isopropanol) was chosen to address the effect of cosolvent bulkiness on pathway competition. The next logical choice in the sequence, *tert*-butanol, was also investigated, but could not be used due to solubility issues.

Increasing the degree of substitution of the alcohol solvent both decreases the α value and increases the β value in a linear fashion. This phenomenon is of course well understood. More aliphatic substitution creates a more electron-rich hydroxyl group, which will behave as a stronger H-bond acceptor and a weaker H-bond donor. It is also well understood that the increase in steric bulk between methanol, ethanol and isopropanol is not linear. The increase in effective “bulkiness” from ethanol to isopropanol is much larger than that from methanol to ethanol.

### 2.1. Increased Elimination with Increased Co-Solvent Bulkiness

The plots in Figure 2 show that the relative amounts of elimination Product **4** increase with increasing cosolvent bulkiness. This can be seen in the “stacking” of lines on each plot. For each IL, the amount of **4** increases between methanol and ethanol and then increases again between ethanol and isopropanol. This was expected. Considering the reaction in its most basic form, we are observing the interaction between a tertiary carbocation and an alcohol. The larger the alcohol, the more likely deprotonation will occur as substitution becomes disfavored due to steric strain.

In looking at the finer details of each plot and comparing them, one can see that there is much more going on in this system than cosolvent bulkiness can explain. Slopes of each plot vary widely. Some increase from methanol to ethanol to isopropanol, whereas others decrease. Still others follow no apparent pattern. To rationalize these results, we must also look at the Kamlet–Taft parameters of hydrogen bond donating and accepting ability, α and β respectively. For any ionic liquid, the cationic component is responsible for hydrogen-bond donating ability (α), and the anionic component is responsible for hydrogen-bond accepting ability (β). Therefore, differences caused by changes in α and β can be isolated with careful selection of IL components. For the remainder of this discussion, we will therefore divide our ILs into two sets: one series in which β values are held constant and α values varied (*i.e.*, ILs with the same anionic component and different cationic components) and one series in which α values are held constant and β values varied (*i.e.*, ILs with the same cationic component and different anionic components).

### 2.2. Ionic Liquids with Varying α Values

Three of the ILs chosen for this study have the same anionic component, [NTf_2_], and differing cationic components: [C_4_C_1_C_1_^2^im], [C_4_pyr] and [C_4_C_1_im]. Plots for these are shown in Figure 2a–c; the slopes of these plots and Kamlet–Taft (KT) parameters are shown in the top portion of Table 1. Notice from Table 1 that β values for these ionic liquids are nearly the same, whereas α values range from 0.38–0.62. Furthermore, each IL α value is lower than each alcohol α value. Therefore, for all of these dual solvent systems, the overall α value of the solvent system decreases as more ionic liquid is added (from left to right along the x-axis in the plots in Figure 2). It should also be noted that the β value decreases, as well, but for this series (since β values of the ILs are nearly identical), the rate of decrease of β is consistent and, so, any effect due to this would cancel out while making comparisons. The changing α value of the system affects whether the alcohol cosolvent is more likely to act as a nucleophile or a base toward the carbocation intermediate **2**. A large α value means that the solvent system is a good hydrogen bond donor. Hydrogen bond donation to the nucleophile causes it to become much “softer” and therefore more nucleophilic/less basic. Decreasing this α value by adding IL therefore causes the nucleophile to become more basic, favoring the elimination pathway. This rational was used to explain the positive slopes for the methanol data [14] and still holds true; most of the slopes reported in Table 1 are positive.

Looking at the difference between IL and alcohol α values gives an idea of the magnitude of this effect (*i.e.*, how dramatically the α value decreases as more IL is added). This was explained in great detail in our previous paper [16]. For example, for a single IL, methanol cosolvent would have the most rapid decrease in α with increasing mole fraction IL (because methanol has the largest α), whereas isopropanol would have the most gradual (because isopropanol has the smallest α). Based on this analysis, we would therefore expect slopes to decrease from methanol to ethanol to isopropanol, while still remaining positive.

In considering these three ILs, we must consider both the effect of this changing α value and the effect of the changing bulkiness of the alcohol cosolvent. Recall that a bulkier alcohol cosolvent favors the elimination pathway due to steric hindrance of the competing substitution pathway. This would lead one to expect an increase in slopes from methanol to ethanol to isopropanol. However, we also rationalized that as the change in α value of the solvent system is decreased (*i.e.*, a small difference in α between IL and cosolvent), one expects to see a more gradual increase in elimination product as the substitution pathway becomes more competitive. This should lead to a decrease in slopes from methanol to ethanol to isopropanol. These two effects are clearly in opposition to one another.

In looking at the data, it is observed that for [C_4_C_1_im][NTf_2_], slopes increase from methanol to ethanol to isopropanol (Figure 2a and Table 1, Entry a). It therefore appears that the bulkiness of the alcohol cosolvent is the major factor affecting the pathway in this case. This IL also has the largest α value of the three and, therefore, closest to the alcohol α values. It is therefore not surprising to see that the changing α value is overridden by the effect of alcohol bulkiness. In contrast, [C_4_C_1_C_1_^2^im][NTf_2_] shows decreasing slopes from methanol to ethanol to isopropanol (Figure 2c and Table 1, Entry c). This IL has the smallest α value of the three, leading to a more dramatic change in α with changing IL mole fraction. We therefore rationalize that for this case, the rapidly decreasing α value overrides cosolvent bulkiness. The third IL, [C_4_pyr][NTf_2_], appears to have found the balance between these two competing factors (Figure 2b and Table 1, Entry b). Slopes are much more consistent between the three alcohol cosolvents.

### 2.3. Ionic Liquids with Varying β Values

Next, we will consider the three ILs that have the same cationic component, [C_4_C_1_im], and differing anionic components: [OTf], [NTf_2_] and [PF_6_]. Plots for these are shown in Figure 2d–f; slopes of these plots and KT parameters are shown in the bottom portion of Table 1. Notice from Table 1 that α values for these ionic liquids are nearly the same, whereas β values range from 0.21–0.46. Again, each IL β value is lower than each alcohol β value. Therefore, for all of these dual solvent systems, the overall β value of the solvent system decreases as more ionic liquid is added (from left to right along the x-axis in the plots in Figure 2). The changing β value of the system also affects whether the alcohol cosolvent is more likely to act as a nucleophile or a base toward the carbocation intermediate **2**. A large β value means that the solvent system is a good hydrogen bond acceptor. Hydrogen bond accepting from the nucleophile causes it to become much more electron rich or “harder” and, therefore, more basic/less nucleophilic. Decreasing this β value by adding IL therefore causes the nucleophile to become more nucleophilic, favoring the substitution pathway.

Again, we must consider the difference in β values between the IL and the cosolvent in order to understand the magnitude of this effect. Since β values increase from methanol to ethanol to isopropanol, isopropanol should demonstrate this effect the most dramatically and should therefore display a smaller slope (a more gradual increase in elimination as it is tempered by the β value’s inclination towards substitution). Based on this analysis, we would expect slopes to decrease from methanol to ethanol to isopropanol (as substitution becomes more favorable). This is in competition with our predictions for cosolvent bulkiness (*i.e.*, increasing slopes from methanol to ethanol to isopropanol). We have also identified a third factor that must be taken into consideration: the bulkiness of the IL anion. Since the three ILs in this series have different anions, we must consider the differences in coordination of these anions to the carbocation intermediate. A bulkier IL anion would hinder the substitution pathway, making elimination more favorable. For our selected anions, bulkiness increases from [OTf] to [NTf_2_] to [PF_6_]. Increasing the amount of ionic liquid could therefore result in more steric hindrance for the substitution pathway, causing elimination to become more favorable. This factor should be most evident in the [PF_6_] IL.

In looking at the data, it is not entirely clear how these three factors work together. For [C_4_C_1_im][OTf] (Figure 2d and Table 1, Entry d), the slopes are all very similar, which would indicate a balance between the three factors. For [C_4_C_1_im][NTf_2_] (Figure 2e and Table 1, Entry e), an obvious trend of increasing slopes is observed, indicating that cosolvent and anion bulkiness are working together to favor the elimination pathway and to override the change in β value of the solvent system. The last IL [C_4_C_1_im][PF_6_] (Figure 2f and Table 1, Entry f), is the most puzzling, as there is no clear trend observed. Specifically, the [C_4_C_1_im][PF_6_]:isopropanol dual solvent system displays an unexpected large negative slope. This would indicate that the effect of the changing β value must be overriding any steric argument, though this does not seem to follow the rest of the data. More likely, another factor may be affecting this particular solvent system. Studies are currently underway to better understand what is happening here.

## 3. Experimental Section

### 3.1. General

The triflate derivative of pivaloin, **1**, was prepared from the corresponding alcohol as previously described [15]. The ionic liquid, 1-butyl-3-methylimidazolium bistrifluoromethane-sulfonamide, [C_4_C_1_im][NTf_2_], was prepared and dried as previously described [12]. Water-insoluble ionic liquids [C_4_C_1_C_1_^2^im][NTf_2_] and [C_4_pyr][NTf_2_] were prepared in an identical fashion. Water-soluble ionic liquids [C_4_C_1_im][PF_6_] and [C_4_C_1_im][OTf] were prepared as previously described [18]. Anhydrous alcohols were purchased and used as-is. 2,6-Lutidine was purchased and used as-is.

### 3.2. Solvolyses

The following procedure is representative for solvolyses involving methanol/ionic liquid dual solvent systems. A solution of 2.67 g dry isopropanol and 1.48 g dry [C_4_C_1_im][NTf_2_] (0.074 mole fraction ionic liquid) was added to a small vial containing 15.9 mg (0.052 mmol, 1 eq) triflate **1** and 12.9 mg (0.120 mmol, 2.3 eq) 2,6-lutidine. The reaction solution was stirred for one minute and transferred to a N_2_-flushed reaction tube. The tube was capped with a rubber septum and placed in a water bath at 45 °C for 19 h. The reaction solution was extracted with three 2-mL portions of hexanes. The combined hexane extracts were washed with water, dried over MgSO_4_ and filtered. The solvent was removed by rotary evaporation. The crude residue was dissolved in chloroform-d and analyzed by ^1^H-NMR (500 MHz). The peaks corresponding to the α-carbonyl protons of **3** and **4** were integrated to determine product ratios.

## 4. Conclusions

For IL:alcohol dual solvent systems, it appears that four factors affect the choice of substitution *vs.* elimination pathways in unimolecular solvolysis reactions. These four factors are the hydrogen bond donating ability of the solvent, α, the hydrogen bond accepting ability of the solvent, β, the bulkiness of the alcohol cosolvent and the bulkiness of the IL anion. It was found, not surprisingly, that increasing the bulkiness of alcohol cosolvent led to increased elimination product. The comparison of ILs with the same anion (and different cations) allowed the comparison between changing α values of alcohol bulkiness. These two factors are in competition, and that competition is swayed by the rate of change of α with increasing IL concentration. The comparison of ILs with the same cation (and different anions) allowed the comparison between three factors: changing β values, alcohol bulkiness and IL anion bulkiness. Trends here were less clear and are under further investigation. In conclusion, the favorability of the substitution and the elimination mechanistic pathways correlate strongly, though not completely, with the Kamlet–Taft solvatochromic parameters of the hydrogen bond donating and accepting ability of the ILs. It is interesting to note that this differs from the results found for bimolecular (S_N_2) substitution reactions [10,11].
